# Heterogeneous regulation of fibroblast growth factor 23 in acute kidney injury, chronic kidney disease, and polycystic kidney disease: mechanisms, diagnostic utility, and clinical implications

**DOI:** 10.3389/fmolb.2026.1764206

**Published:** 2026-02-17

**Authors:** Xiaohua Hu, Bo Yang, Haimin Chen, Min Min, Nanmei Liu, Cheng Xue

**Affiliations:** 1 Department of Nephrology, Zhabei Central Hospital of Jing’an District, Shanghai, China; 2 Department of Nephrology, Naval Medical Center, Naval Medical University, Shanghai, China; 3 Department of Hematology and Oncology, Zhabei Central Hospital of Jing’an District, Shanghai, China; 4 Department of Nephrology, Shanghai Changzheng Hospital, Naval Medical University, Shanghai, China

**Keywords:** acute kidney injury, autosomal dominant polycystic kidney disease, chronic kidney disease, FGF23, heterogeneous regulation

## Abstract

Fibroblast Growth Factor 23 (FGF23) is a bone-derived hormone regulating phosphate and vitamin D metabolism, now recognized as a dynamic biomarker across acute and chronic kidney disorders. Elevated FGF23 is a hallmark of chronic kidney disease (CKD), but also rises acutely in acute kidney injury (AKI) and appears disproportionately high in autosomal dominant polycystic kidney disease (ADPKD), underscoring condition-specific regulation. This review explores the correlation and heterogeneity of FGF23 expression in AKI, CKD, and ADPKD, highlighting shared and divergent mechanisms and the diagnostic and therapeutic implications. We summarize FGF23 expression kinetics in each condition, elucidate known and proposed molecular drivers of its elevation, and discuss how FGF23 serves as a unifying yet disease-divergent marker in renal pathology. In AKI, inflammation, ischemia, and acute metabolic stress drive a rapid FGF23 surge, whereas in CKD, phosphate retention and Klotho deficiency promote a sustained, maladaptive FGF23 elevation. ADPKD shows early FGF23 increases independent of glomerular filtration rate (GFR), potentially due to ectopic production (liver and cysts) and unique tubular defects. Clinically, FGF23 has emerged as an indicator of disease severity and outcomes in these contexts: it can signal early AKI and predict progression, is a strong prognostic factor for mortality and cardiovascular complications in CKD, and correlates with cystic disease burden and kidney growth in ADPKD. We also examine FGF23’s systemic effects (notably on cardiovascular remodeling) and potential therapeutic targets, from modulating phosphate balance and iron metabolism to novel interventions in development. Understanding the nuanced regulation of FGF23 across acute injury, chronic degeneration, and genetic kidney disease provides insight into acute-chronic disease intersections and guides precision diagnostics and therapies for improved patient outcomes.

## Introduction

Fibroblast Growth Factor 23 (FGF23) is a bone-derived phosphaturic hormone that plays a critical role in phosphate and vitamin D homeostasis (Shimada et al., 2004). Secreted primarily by osteocytes and osteoblasts, FGF23 acts on the kidney via its co-receptor Klotho and FGF receptor-1 (FGFR1) to promote urinary phosphate excretion and suppress 1,25-dihydroxyvitamin D synthesis (Rroji et al., 2023). Under healthy conditions, FGF23 helps maintain mineral balance by reducing serum phosphate and parathyroid hormone (PTH) levels when dietary phosphate is high, or calcitriol is elevated (Isakova et al., 2011a). In pathological states, however, FGF23 regulation is markedly altered–especially in the context of kidney dysfunction.

Elevated circulating FGF23 has emerged as both a biomarker and potential mediator of disease in acute and chronic renal conditions (Lu et al., 2023). Acute kidney injury (AKI), chronic kidney disease (CKD), and autosomal dominant polycystic kidney disease (ADPKD) each exhibit distinct profiles of FGF23 dysregulation despite all featuring renal involvement. These differences reflect the heterogeneous pathophysiology between acute injury, chronic degenerative disease, and genetic structural disease. At the same time, FGF23’s presence in all three suggests it may be a unifying indicator of “renal stress,” albeit via disease-specific mechanisms. This aligns with the broader theme of exploring how acute and chronic conditions intersect: FGF23 provides a lens to examine how an acute insult and chronic diseases can share common pathways dyet diverge in their regulation and outcomes.

Notably, CKD has long been associated with progressively rising FGF23 levels even in early stages (Isakova et al., 2011a). More recent evidence shows FGF23 can increase rapidly in AKI–often within hours of injury–pointing to regulatory triggers beyond the classical slow rise due to phosphate retention (Zhang and Qin, 2023). Furthermore, in a genetic disease like ADPKD, FGF23 elevations occur early, implicating alternative sources or stimuli unrelated to the usual CKD mineral disturbances (Pavik et al., 2011). These findings underscore that while FGF23 elevation is a common thread, its drivers and implications are context-dependent.

In this review, we discuss FGF23 regulation across three paradigmatic kidney conditions–AKI, CKD, and ADPKD. We detail how FGF23 levels change in each setting, the underlying mechanisms, and the clinical significance of these patterns.

## FGF23 expression pattern in AKI, CKD, and ADPKD

**FIGURE 1:**
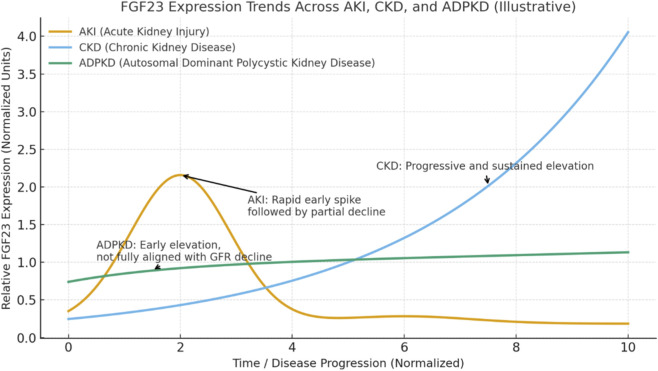
Illustrative trajectories of plasma FGF23 expression in AKI, CKD, and ADPKD. FGF23 shows distinct kinetics across kidney diseases. In AKI, there is a rapid transient spike during the early phase followed by partial normalization. In CKD, FGF23 rises progressively with disease progression, reflecting phosphate retention and decreased renal clearance. In ADPKD, FGF23 is elevated even in early stages and increases slowly thereafter, indicating disease-specific dysregulation not fully explained by eGFR decline. The x-axis represents a normalized disease progression continuum rather than absolute chronological time, The x-axis represents a normalized disease progression continuum rather than absolute chronological time. This schematic is intended to highlight relative temporal patterns and disease-stage–dependent differences in FGF23 regulation among acute and chronic kidney diseases.

AKI is characterized by a swift rise in circulating FGF23 that can occur within hours to days of the inciting injury (Zhang and Qin, 2023). The first clinical hint of this phenomenon came from an observation of extremely high FGF23 in a patient with AKI due to rhabdomyolysis (Leaf et al., 2010). Subsequent studies in both patients and animal models have confirmed that FGF23 spikes early in AKI, often preceding significant changes in serum phosphate or traditional kidney function markers (Zhang and Qin, 2023). For example, critically ill patients who develop AKI show markedly elevated FGF23 levels relative to those who do not, and infants after cardiac surgery demonstrate that FGF23 measured postoperatively can predict imminent severe AKI (de Oliveira Neves et al., 2019; Volovelsky et al., 2018). Figure 1 illustrates trajectories of plasma FGF23 expression in AKI, CKD, and ADPKD.

Both intact FGF23 (iFGF23) and C-terminal fragments (cFGF23) rise in AKI. In many studies, the C-terminal assay registers the most dramatic increases (Sun et al., 2021). Clinically, these elevations correlate with injury severity and outcomes: higher FGF23 in AKI associates with need for dialysis, progression to CKD, and increased in-hospital mortality (Lu et al., 2023; Zhang and Qin, 2023). Interestingly, the magnitude of FGF23 elevation in AKI can exceed what is observed in CKD patients with similar creatinine levels, suggesting non-renal factors contribute to the acute surge (Christov et al., 2013). This transient spike typically declines as renal function recovers, distinguishing it from the sustained high FGF23 plateau seen in CKD (Hanudel et al., 2016).

It is important to note that reduced kidney function will elevate FGF23 by impairing its clearance. However, in AKI, the FGF23 rise often outpaces the modest changes in glomerular filtration, implying increased production plays a major role (Christov et al., 2013). Indeed, AKI triggers an active endocrine response: rising FGF23 appears to be part of the acute “stress” response to injury, rather than a simple consequence of phosphate retention. The clinical utility of measuring FGF23 in AKI is under investigation. Early data suggest it could serve as an early biomarker of AKI, potentially before creatinine rises, and a prognostic marker: one study found AKI patients who later developed CKD had significantly higher FGF23 at the time of their AKI than those who recovered fully (Lu et al., 2023). Thus, the expression pattern in AKI is one of a rapid, pronounced but transient FGF23 elevation, reflecting acute pathophysiological stress and portending poorer outcomes when especially high.

In CKD, FGF23 elevation is a well-established and early event in the progression of disease. Unlike the transient spike in AKI, CKD patients experience a chronic, progressive rise in FGF23 levels that can reach extraordinarily high values in advanced stages. Notably, plasma FGF23 begins to increase as early as CKD stage 2 – even before any overt decline in GFR, hyperphosphatemia, or rise in PTH is detected (Ix et al., 2010). A landmark study by Isakova et al. showed that in CKD stages 2–4, median FGF23 was significantly elevated despite normal serum phosphate and PTH, and FGF23 rose sharply with even mild eGFR reduction, preceding other mineral markers (Isakova et al., 2011a). Thus, FGF23 is a sensitive early biomarker of disordered phosphate metabolism in CKD.

As kidney function deteriorates further, FGF23 levels climb exponentially. By CKD stage 4–5, FGF23 may be tens-to hundreds-fold above normal, and in end-stage renal disease (ESRD) on dialysis, FGF23 can reach levels 100–1,000 times higher than in healthy individuals (Vogt et al., 2019). Studies have documented FGF23 concentrations >5,000 RU/mL in dialysis patients (normal ∼50 RU/mL), reflecting this dramatic accumulation (Jean et al., 2009). Both intact FGF23 and C-terminal fragments accumulate in CKD, although intact FGF23 remains the main driver of endocrine effects (David et al., 2016). The chronic nature of FGF23 elevation in CKD contrasts with the transient AKI pattern–FGF23 in CKD is persistently high and continues to rise over time, contributing to a chronic “FGF23 load” on various tissues.

The pattern of FGF23 increase in CKD can be summarized as follows: it starts early (stage 2–3 CKD) with relatively modest elevations (often 2–5 fold), then accelerates as GFR falls below ∼30 mL/min, and reaches extreme levels in late stage 5 CKD and dialysis (Komaba, 2023). This trajectory often outpaces the rise in serum phosphate. In fact, normophosphatemia can be maintained until late CKD precisely because FGF23 and PTH increase to compensate (Rodelo-Haad et al., 2025). Eventually, however, the adaptive capacity is exceeded, and hyperphosphatemia appears alongside very high FGF23. The persistent elevation of FGF23 in CKD is a key distinguishing feature from AKI and has significant downstream consequences.

Clinically, FGF23 in CKD is associated with many of the complications of mineral and bone disorder (CKD-MBD). It correlates inversely with residual renal function and directly with phosphate levels as CKD progresses (Komaba, 2023). Since it rises so early, high FGF23 can serve as a warning sign in CKD patients who still have normal phosphate, indicating that phosphate balance is being maintained only by a stressed hormonal response. It also means therapies aimed at reducing CKD-MBD risk might be considered even before overt hyperphosphatemia, guided in part by FGF23 trends. In summary, the expression pattern of FGF23 in CKD is a steadily increasing, sustained elevation that begins early and culminates in potentially 1000-fold excess levels in ESRD, marking it as a hallmark hormonal change of chronic kidney failure (Vogt et al., 2019).

ADPKD is a hereditary condition characterized by bilateral renal cyst formation, often leading to CKD over decades. Intriguingly, patients with ADPKD exhibit elevated FGF23 levels at relatively early disease stages, often out of proportion to their kidney function impairment (Pavik et al., 2011). Several studies have noted that ADPKD patients with preserved GFR can have significantly higher plasma FGF23 compared to non-cystic CKD patients at similar GFR levels (Pavik et al., 2011; Pavik et al., 2012; Yildiz et al., 2014). In other words, FGF23 is disproportionately elevated in ADPKD. Pavik et al. first reported that even ADPKD individuals with normal kidney function had elevated FGF23 and a tendency toward low-normal phosphate, indicating a renal phosphate “leak” (6). This pattern contrasts with typical CKD and with AKI. In ADPKD, the elevation appears *early* and persists throughout the disease course, even before classical CKD-mineral disturbances manifest.

Both intact and C-terminal FGF23 levels are elevated in ADPKD, though their relative proportions may vary. Some ADPKD patients show high total FGF23 with only moderate intact FGF23 elevation, suggesting increased FGF23 production accompanied by enhanced cleavage (Hanudel et al., 2019a). Others, especially as GFR falls, have high intact FGF23 as well. The “FGF23 signature” of ADPKD includes: Normal serum phosphate or even hypophosphatemia in early stages, despite high FGF23 (6). - Elevated FGF23 even when eGFR is normal or mildly reduced, indicating disease-specific drivers beyond just kidney function (Pavik et al., 2011). - A renal threshold for phosphate reabsorption (TmP/GFR) that is lower than expected given the level of FGF23 (Righini et al., 2024). - Progressive rise in FGF23 as kidney volume increases and CKD progresses, eventually overlapping with levels seen in other CKD causes at later stages.

A recent large cohort study (DIPAK, 2024) found that about 59% of ADPKD patients had evidence of renal phosphate wasting (low TmP/GFR <0.8 mmol/L), and those patients tended to have higher FGF23 levels and more severe disease progression (Xue et al., 2024). Importantly, ADPKD patients with phosphate wasting had faster eGFR decline and a higher risk of reaching kidney failure compared to those without phosphate leak (Xue et al., 2024). This suggests that FGF23 elevation and its phosphate-wasting effects may be markers of a more aggressive cystic disease phenotype.

## Mechanisms driving FGF23 elevation in AKI

The acute FGF23 surge in AKI is thought to result from both increased production and diminished clearance of the hormone (Zhang and Qin, 2023). Several interrelated mechanisms have been proposed to explain this pronounced rise.

### Inflammation and cytokine signaling

AKI is often accompanied by a systemic inflammatory response. Pro-inflammatory cytokines such as interleukin-6 (IL-6), tumor necrosis factor α (TNF-α), and IL-1β can stimulate FGF23 gene expression in bone. IL-6 in particular has been shown to directly induce FGF23 transcription via STAT3 signaling in osteocytes (Durlacher-Betzer et al., 2018). In AKI patients and models, IL-6 levels rise sharply; this has been causally linked to FGF23 upregulation. For instance, Radhakrishnan et al. demonstrated that AKI in mice triggers IL-6, which in turn induces hepatic expression of the nuclear receptor ERRγ, driving FGF23 production in the liver (Radhakrishnan et al., 2021). In summary, acute inflammation during AKI provides a potent stimulus for FGF23 production, both in bone and ectopically (e.g., liver), through cytokine-driven transcriptional pathways (Czaya and Faul, 2019).

### Acute tissue ischemia and metabolic signals

Ischemia-reperfusion injury in AKI leads to cellular metabolic stress. One novel pathway involves glycerol-3-phosphate (G-3-P), a glycolytic byproduct that accumulates during renal ischemia (Zhou et al., 2022). Simic et al. discovered that injured kidneys release G-3-P into circulation; in bone, G-3-P is converted to lysophosphatidic acid, which activates osteocyte LPA_1 receptors and markedly increases FGF23 secretion (Simic et al., 2020). This mechanism provides a direct link between acute tubular injury and remote bone endocrine function. Hypoxia-inducible factors (HIFs) activated in ischemic kidneys may also play a role in FGF23 regulation, as they do in conditions like iron deficiency (Edmonston and Wolf, 2020). Thus, AKI-related hypoxia and metabolic reprogramming can send *FGF23-inductive signals* to bone.

### Reduced renal clearance

Even a short-lived decline in GFR will reduce the kidney’s ability to catabolize and excrete FGF23 (11). FGF23 is normally filtered and possibly degraded by kidneys; hence AKI can cause retention of circulating FGF23 (Mace et al., 2015). Studies suggest this is a contributing factor but not the sole explanation, given the outsized increase of FGF23 relative to the degree of GFR drop in many AKI cases (Zhang and Qin, 2023). Nonetheless, impaired clearance likely augments the hormone’s accumulation during AKI.

### Klotho shedding

AKI is known to cause an acute loss of Klotho through renal shedding and reduced expression (Hu et al., 2010). Diminished Klotho might paradoxically feedback to drive FGF23 higher, as tissues sense FGF23 resistance. Moreover, Klotho itself has renoprotective effects; its loss could exacerbate injury. Preclinical studies show exogenous Klotho can ameliorate AKI severity (Hu et al., 2021), suggesting a complex interplay where FGF23 rises and Klotho falls during AKI, each potentially influencing the other’s levels.

### Bone marrow and hormonal crosstalk

AKI is associated with early disturbances in iron handling and erythropoiesis (van Swelm et al., 2020). Inflammatory AKI raises hepcidin, causing functional iron deficiency; in turn, iron deficiency stabilizes HIF-1α in osteocytes, which increases FGF23 transcription (Rroji et al., 2023). Additionally, high doses of erythropoietin (EPO) given in acute illness can acutely raise FGF23 production (with increased cleavage, so intact hormone changes may be muted) (Martínez-Heredia et al., 2024). While these factors are more established in chronic disease, they may also contribute if AKI is accompanied by anemia management and inflammation.

### Mechanisms behind FGF23 upregulation in CKD

Multiple interlocking mechanisms drive the sustained upregulation of FGF23 in CKD. These can be viewed as initially adaptive responses to declining renal function that later become maladaptive contributors to pathology.

### Phosphate retention

Reduced nephron mass in CKD leads to decreased phosphate excretion (Rodelo-Haad et al., 2025). Even before serum phosphate rises, the body senses phosphate “loading,” which stimulates osteocytes to secrete more FGF23 to enhance phosphaturia (Pereira et al., 2009). This is a key homeostatic driver: FGF23 increases in an attempt to maintain neutral phosphate balance despite falling GFR (Rodelo-Haad et al., 2025). Early CKD studies showed an inverse relationship between renal phosphate clearance and FGF23 levels (Komaba and Fukagawa, 2009). However, as CKD advances, even massive FGF23 elevations cannot prevent hyperphosphatemia because the kidneys simply cannot excrete enough phosphate (Vogt et al., 2019). In essence, chronic phosphate retention is the fundamental stimulus for chronic FGF23 elevation. Notably, interventions like phosphate binders or low-phosphate diet can reduce FGF23 levels in CKD, underscoring phosphate as a driving factor (Zhao et al., 2022; Cozzolino et al., 2013).

### Klotho deficiency and FGF23 resistance

The failing kidney produces less Klotho (Lu and Hu, 2017). Declining Klotho expression is a hallmark of CKD and has two major effects on FGF23 dynamics. First, low renal Klotho makes the kidney less responsive to FGF23, meaning phosphate excretion does not increase appropriately even as FGF23 rises (Vogt et al., 2019). This resistance causes a vicious cycle: sensing ineffectiveness, osteocytes secrete even more FGF23 in compensation (Vogt et al., 2019). Second, Klotho deficiency extends to the parathyroid glands and perhaps other tissues, where the FGFR–Klotho complex is needed for FGF23’s hormonal actions. In advanced CKD, parathyroid glands have reduced Klotho/FGFR1, rendering them less sensitive to FGF23’s usual PTH-suppressing effect. Consequently, FGF23 loses its ability to inhibit PTH in late CKD–high FGF23 and high PTH coexist because the signal to the parathyroid is resisted (Czaya and Faul, 2019). This contributes to secondary hyperparathyroidism. Overall, early Klotho loss in CKD both triggers a maladaptive FGF23 surge and nullifies some of FGF23’s regulatory feedback, exacerbating mineral imbalance (Nakagawa and Komaba, 2024).

### Disordered vitamin D and PTH feedback

As FGF23 rises, it suppresses 1,25-dihydroxyvitamin D, which lowers calcium absorption and contributes to hypocalcemia and hyperparathyroidism (Bergwitz and Jüppner, 2010). Meanwhile, by late CKD, PTH is markedly elevated (Nakagawa and Komaba, 2024). PTH itself may influence FGF23: studies suggest PTH can stimulate FGF23 production, and conversely, high FGF23 normally suppresses PTH(34). In CKD, this cross-talk is disrupted. The net effect is a feed-forward loop: declining calcitriol and rising PTH both drive more FGF23 secretion (Sharma and Ix, 2023). Thus, the normal endocrine axes are skewed, with FGF23, PTH, and vitamin D locked in a pathological imbalance.

### Chronic inflammation and oxidative stress

CKD is a state of persistent low-grade inflammation (Czaya and Faul, 2019; Oberg et al., 2004). Circulating levels of IL-6, TNF-α, and other cytokines are often elevated in CKD patients, especially as GFR falls. These inflammatory mediators can chronically stimulate FGF23 production in bone (Munoz Mendoza et al., 2012). Moreover, there is evidence that FGF23 itself can exacerbate inflammation: FGF23 signalingparticularly via FGFR4may promote production of inflammatory cytokines in certain contexts (Singh et al., 2016). A cycle may thus form where inflammation raises FGF23, which in turn feeds back to worsening inflammation and tissue injury, contributing to CKD progression (Czaya and Faul, 2019). Oxidative stress, often linked with inflammation, can activate osteocyte signaling pathways (like NF-κB or HIF) that upregulate FGF23 transcription (Rodelo-Haad et al., 2025; Czaya and Faul, 2019). In summary, the inflammatory milieu of CKD adds a smoldering stimulant to FGF23 synthesis beyond the direct mineral metabolism factors.

### Iron deficiency and erythropoietic stimuli

CKD patients frequently develop anemia and functional iron deficiency (Courbon et al., 2020). This has a complex effect on FGF23 regulation. Iron deficiency in bone triggers increased FGF23 gene transcription via HIF-1α, but it also increases FGF23 cleavage, so intact FGF23 may not rise proportionally unless iron deficiency is accompanied by inflammation or CKD (Rroji et al., 2023). In CKD, there is both increased production and reduced degradation of FGF23, so iron deficiency tends to raise total and intact FGF23. Treatment with erythropoiesis-stimulating agents (ESAs) like EPO can acutely raise total FGF23, but also enhance FGF23 cleavage–resulting in transiently higher C-terminal fragment levels without a large spike in intact hormone (Czaya and Faul, 2019). Notably, in CKD animals that have impaired FGF23 cleavage, EPO injection does elevate intact FGF23 significantly (Hanudel et al., 2019b). This suggests that in advanced CKD, EPO therapy could contribute to higher active FGF23. On the flip side, FGF23 itself affects iron and erythropoiesis: it suppresses renal EPO production and increases hepatic hepcidin via inflammatory cytokine induction (Czaya and Faul, 2019; Coe et al., 2014). This bidirectional relationship ties anemia management to FGF23 levels. Clinically, correcting iron deficiency in CKD has been shown to lower FGF23 levels in many cases, improving both anemia and reducing FGF23’s potential toxicity (Martínez-Heredia et al., 2024; Courbon et al., 2020). Thus, iron/EPO disturbances in CKD represent another layer of FGF23 regulation.

In combination, these mechanisms paint a picture of FGF23 regulation in CKD as initially compensatory but eventually pathologic. Phosphate retention provides the initial push; Klotho loss removes the brakes; disordered feedback with PTH/vitamin D, chronic inflammation, and anemia-related factors all further fuel FGF23 overproduction. The result is a hormone that becomes drastically elevated and part of the CKD pathophysiology itself. Addressing these upstream drivers–phosphate burden, Klotho deficiency, inflammation, and iron deficiency–is central to managing CKD-MBD and could modulate FGF23 levels.

## Mechanisms behind FGF23 elevation in ADPKD

The drivers of FGF23 dysregulation in ADPKD appear to be a combination of factors seen in CKD and factors unique to the cystic disease environment. Key proposed mechanisms include.

### Ectopic production (hepatic FGF23)

Unlike AKI or ordinary CKD where FGF23 originates mainly from bone, ADPKD seems to feature extra-osseous FGF23 production, particularly in the liver. ADPKD patients commonly have polycystic liver disease. Bienaimé et al. showed that severely polycystic livers express FGF23 mRNA and protein, and that circulating FGF23 levels in ADPKD correlated with liver cyst volume (Bienaimé et al., 2018). This suggests the liver cyst epithelium can produce FGF23, contributing to systemic levels. The mechanism may involve the polycystin-1/2 mutation effect on the liver or the local environment of cystic liver tissue. Ectopic hepatic production helps explain why some ADPKD patients have high FGF23 even when their kidney function is relatively intact–an additional source independent of renal clearance. Thus, ADPKD represents a scenario where FGF23 regulation is not solely bone-kidney; the liver becomes an endocrine organ for FGF23 (Hanudel et al., 2019a).

### Cystic kidney production and local hypoxia

There is evidence that FGF23 is produced within cystic kidneys themselves. Animal models of PKD have shown cyst-lining cells in the kidney expressing FGF23 mRNA and protein (Spichtig et al., 2014). The microenvironment in polycystic kidneys is characterized by regional ischemia, hypoxia (Theodorakopoulou et al., 2019), and inflammation (Mun and Park, 2016). Hypoxia-inducible factor 1α (HIF-1α) is known to upregulate FGF23 transcription by binding hypoxia-response elements in the FGF23 gene (Hanudel et al., 2019a). ADPKD cysts likely experience hypoxia, which could induce local FGF23 production. In a human ADPKD study, one patient with extensive liver cysts had extraordinarily high FGF23 levels and evidence of bone FGF23 expression, supporting the concept that cystic disease can drive FGF23 (Hanudel et al., 2019a). Chronic inflammation in the cystic kidney may also contribute, similar to CKD. Therefore, cystic remodeling of organs in ADPKD provides a nidus for FGF23 production through hypoxia and inflammatory signaling.

### Early klotho deficiency and FGF23 resistance

ADPKD kidneys exhibit reduced Klotho expression even at early stages (Pavik et al., 2012). Akiyama et al. found that ADPKD patients had lower soluble Klotho levels than non-ADPKD CKD controls at the same eGFR, and higher FGF23 levels, consistent with relative FGF23 resistance (Akiyama et al., 2017). Low Klotho in ADPKD would make the kidney less responsive to FGF23’s phosphate-wasting signal, potentially causing an even greater increase in FGF23 production as compensation. This mechanism mirrors CKD but is noteworthy in ADPKD because Klotho deficiency appears out of proportion to GFR. FGF23 resistance might also manifest as an inappropriately normal or high TmP/GFR despite high FGF23 (Gitomer et al., 2021) – in other words, the phosphate reabsorption in ADPKD kidneys might be higher than expected for the level of FGF23, because of Klotho loss. The net effect is a feedback loop: cystic kidneys are “deaf” to FGF23, phosphate excretion is impaired relative to FGF23 levels, leading osteocytes to further ramp up FGF23 secretion.

### Tubular dysfunction and phosphate wasting

Paradoxically, many ADPKD patients waste phosphate (Xue et al., 2024). This could be due to specific proximal tubular defects or FGFR resistance patterns in the kidney. ADPKD mutations in PKD1/PKD2 affect cilia and cellular signaling, possibly altering how tubules handle minerals. Some hypothesize that primary cilia dysfunction in osteocytes or kidney cells could dysregulate FGF23 production or response (Righini et al., 2024; Mekahli and Bacchetta, 2013). Additionally, as cysts expand, normal nephron segments may become less responsive or structurally altered, leading to impaired phosphate reabsorption. The high copeptin levels in ADPKD and possible differences in tubular transporters might contribute to a lower phosphate reabsorption threshold. The DIPAK study mentioned earlier suggests that a portion of ADPKD patients have an intrinsic proximal tubular defect causing phosphate leak, which correlates with disease severity (Xue et al., 2024).

### FGF23 cleavage abnormalities

Some research indicates that ADPKD might influence the processing of FGF23. Bienaimé et al. noted that ADPKD patients with preserved GFR had a disproportionately high C-terminal FGF23 relative to intact FGF23, suggesting enhanced cleavage of FGF23 into fragments (Bienaimé et al., 2018). This could be due to upregulation of proteases (like furin) in the liver or bone triggered by ADPKD-related factors, or perhaps an effect of the PKD gene on FGF23-producing cells. If FGF23 is being cleaved more, it could create a scenario of high total FGF23 but moderated intact levels in early disease. As ADPKD progresses and CKD sets in, cleavage might decrease, leading to a sharp rise in intact FGF23. Such complexities mean that interpreting FGF23 levels in ADPKD might require understanding the fragment composition. Regardless, altered post-translational processing is another facet of how ADPKD can uniquely modulate FGF23 regulation (Hanudel et al., 2019a; Bienaimé et al., 2018).

In essence, ADPKD’s effect on FGF23 is multi-factorial: part CKD-like, part unique (cystic liver and kidney producing FGF23, early Klotho loss, phosphate handling quirks). The heterogeneity even among ADPKD patients is notable–some with early high FGF23 and phosphorus wasting, others not until later. This likely reflects genetic and structural variability.

## Diagnostic and clinical implications in AKI

The pronounced FGF23 response in AKI carries several clinical implications. Firstly, FGF23 may serve as an early diagnostic biomarker or risk stratifier in acute settings. Traditional markers like creatinine lag behind actual kidney injury; by contrast, FGF23 can increase within hours. Studies in cardiac surgery patients have shown that postoperative FGF23 elevations preceded clinical AKI diagnosis and were predictive of its severity (Volovelsky et al., 2018; Leaf et al., 2016). In critically ill adults and children, high FGF23 levels correlated with development of AKI and higher mortality risk (Leaf et al., 2018; Hanudel et al., 2019c). Thus, measuring FGF23 in emergency or intensive care contexts might identify patients at high risk of AKI or poor outcomes, enabling earlier interventions.

Secondly, FGF23 might play a pathogenic role in AKI outcomes, not just be a bystander. There is emerging evidence that FGF23 can directly affect injured kidneys and other organs. For example, experimental studies have found that FGF23 can activate *injury-primed* renal fibroblasts via FGFR4, amplifying TGF-β signaling and fibrosis (Smith et al., 2017). In mice with acute kidney damage, excess FGF23 promoted renal fibrogenesis, whereas blocking FGF receptors mitigated post-AKI fibrosis (Lu et al., 2023). This suggests that extremely high FGF23 during AKI could contribute to subsequent kidney scarring and the well-documented phenomenon of AKI-to-CKD progression. High FGF23 may also drive acute cardiac dysfunction: FGF23 can induce left ventricular hypertrophy and impair heart function via FGFR4 activation in cardiomyocytes (Rroji et al., 2023). AKI is often accompanied by acute heart failure, and FGF23 is a candidate mediator linking acute renal injury to cardiac stress (Mattinzoli et al., 2023). In short, FGF23 is not only a marker but possibly a *mediator* of the multi-organ complications of AKI.

Thirdly, these insights open the question of therapeutic targeting. While no therapy currently exists specifically to modulate FGF23 in AKI, some indirect approaches show promise in experimental models. Management of hyperphosphatemia blunted the FGF23 surge and improved survival in a mouse model of AKI (Hamid et al., 2024). In that 2024 study, dietary phosphate restriction prevented metabolic acidosis and markedly reduced FGF23 levels and mortality following folic acid-induced AKI (Hamid et al., 2024). This suggests controlling phosphate burden during AKI may reduce FGF23-driven toxicity. Likewise, provision of soluble Klotho or anti-cytokine therapies could theoretically temper the FGF23 spike and its consequences, though these remain speculative. At present, the clinical focus is on using FGF23 as a prognostic indicator in AKI, identifying patients who might benefit from closer monitoring or early dialysis. Whether modulating FGF23 can improve AKI outcomes is an area for future research. Any intervention must be cautious, as FGF23 elevation in AKI might also have adaptive aspects.

## Clinical implications and potential interventions in CKD

FGF23’s chronic elevation in CKD has far-reaching clinical implications, making it both a biomarker of risk and a potential therapeutic target.

### Prognostic marker for mortality and morbidity

High FGF23 is one of the strongest known independent predictors of adverse outcomes in CKD. Large cohort studies have shown that CKD patients with elevated FGF23 have higher risks of death, cardiovascular events, and progression to end-stage renal disease (Kendrick et al., 2011; Isakova et al., 2011b). For instance, in a study of patients with CKD stages 4–5, FGF23 was a significant predictor of progression to dialysis and mortality, even after adjusting for eGFR and phosphate (Kendrick et al., 2011). Another cohort (CKD stages 2–3) found FGF23 levels were independently associated with faster kidney function decline and greater all-cause mortality (Isakova et al., 2011b). These findings have been consistent: each increase in FGF23 correlates with worse survival. The prognostic power of FGF23 is comparable to or stronger than traditional factors like phosphate or PTH. Therefore, FGF23 is increasingly recognized as a key risk stratifier in CKD, potentially guiding how aggressively to manage a patient’s mineral disorder or cardiovascular risk factors.

### Cardiovascular complications

Chronic high FGF23 has direct toxic effects on the cardiovascular system. In CKD, elevated FGF23 is strongly linked to left ventricular hypertrophy (LVH), arterial stiffness, and vascular calcification (Vogt et al., 2019; Martínez-Heredia et al., 2024; Courbon et al., 2020). Mechanistic studies by Faul et al. showed that FGF23 acts on cardiomyocytes via FGFR4 to induce hypertrophic signaling, activating the PLCγ–calcineurin–NFAT pathway and leading to LVH (Faul et al., 2011). This is supported by animal models: rodents given FGF23 develop cardiac hypertrophy, whereas FGFR4 blockade can prevent it even with high phosphate diet (Grabner et al., 2017). Clinically, CKD patients with higher FGF23 levels have greater left ventricular mass and a higher prevalence of heart failure. FGF23 also may contribute to vascular dysfunction–it has been implicated in promoting arterial stiffness and possibly enhancing calcium deposition in vessels (Vogt et al., 2019; Martínez-Heredia et al., 2024). Additionally, FGF23 can activate the renin-angiotensin-aldosterone system and increase sodium retention via effects on the Na-Cl cotransporter, thereby worsening hypertension (Vogt et al., 2019). These multiple pathways qualify FGF23 as a “cardiovascular toxin” in CKD. The recognition of FGF23’s role in uremic cardiomyopathy has prompted interest in therapeutically lowering FGF23 or blocking its cardiac receptors to improve outcomes.

### Bone and mineral disorders

FGF23 is a central player in CKD-mineral and bone disorder (CKD-MBD). By suppressing calcitriol and driving secondary hyperparathyroidism, high FGF23 contributes to renal osteodystrophy and bone fragility in CKD (Komaba, 2023). Some CKD patients develop an adynamic bone disease partly due to oversuppression of PTH by treatments, leading to low bone turnover (Bover et al., 2014). Conversely, early in CKD, high FGF23 with normal PTH can indicate subtle bone mineral abnormalities even before overt changes in bone density or turnover markers (Vogt et al., 2019; Ott, 2012). Monitoring FGF23 might help identify patients at risk for skeletal complications or who might benefit from interventions like vitamin D analogues or calcimimetics (Stubbs et al., 2007).

### Progression of kidney disease

There is emerging evidence that FGF23 might worsen kidney fibrosis and disease progression (Isakova et al., 2011b; Kosugi et al., 2025), as mentioned earlier in the AKI-to-CKD context. In CKD models, excess FGF23 signaling, especially via FGFR4, can activate pro-fibrotic pathways (Lu et al., 2023). Whether lowering FGF23 would slow CKD progression in humans is not yet proven, but it remains an intriguing hypothesis. It has been observed that interventions reducing FGF23 can associate with stabilization of kidney function, though cause-effect is hard to establish given confounders (Courbon et al., 2020; Damasiewicz et al., 2011).

### Therapeutic interventions

Managing FGF23 in CKD is complex because FGF23 elevation is a consequence of adaptive responses. Directly targeting FGF23 could normalize FGF23 levels but at the cost of precipitating hyperphosphatemia and calcitriol increases, which could accelerate vascular calcification (Vogt et al., 2019). In fact, animal studies confirm that indiscriminate FGF23 blockade in CKD leads to dangerous hyperphosphatemia and greater vascular calcifications (Shalhoub et al., 2012). Therefore, the consensus is that indirect strategies are safer: reducing the stimuli for FGF23 or blocking its harmful effects rather than FGF23 itself. Key approaches include.

### Phosphate control

Dietary phosphate restriction, phosphate binders, and optimized dialysis are mainstays, which can reduce FGF23 by 20%–50% in CKD patients (Vogt et al., 2019; Zhao et al., 2022). Notably, iron-based binders (ferric citrate) both bind phosphate and treat iron deficiency, yielding significant FGF23 reductions and improved anemia (Zhao et al., 2022; Agoro and White, 2022).

### Vitamin D and calcimimetics

Active vitamin D sterols can suppress PTH, but they raise FGF23. Calcimimetics reduce PTH and calcium × phosphate product, which might indirectly lower FGF23 or at least mitigate its drivers (Koizumi et al., 2012). Trials have not primarily focused on FGF23, but one analysis (EVOLVE trial) noted that patients with greater FGF23 reductions had better outcomes (Moe et al., 2015).

### Iron supplementation and anemia management

Correcting iron deficiency in CKD can lower FGF23, especially C-terminal fragments, by reducing HIF-driven production and perhaps increasing FGF23 cleavage (Agoro and White, 2022). Intravenous iron, interestingly, acutely raises intact FGF23 by reducing cleavage, but over time, both IV and oral iron reduce total FGF23 and improve anemia (Takkavatakarn et al., 2022). New therapies like HIF-prolyl hydroxylase inhibitors (used for anemia) have been shown in animal studies to reduce FGF23 levels while improving iron utilization (Noonan et al., 2020). Keeping hemoglobin in the target range with minimal ESA dosing may also stabilize FGF23 levels (Agoro and White, 2022).

### Targeted blockade of FGF23’s effects

Rather than neutralize FGF23, an alternative is to block its deleterious pathways. One example is FGFR4 inhibitors to prevent cardiac hypertrophy. Experimental studies show that FGFR4 blockade can protect the heart from FGF23-induced hypertrophy without affecting the kidney which primarily uses FGFR1/Klotho for FGF23 (Grabner et al., 2017).

### Klotho augmentation

Restoring Klotho levels could improve FGF23 sensitivity and mitigate the compensatory FGF23 hypersecretion. Animal models of CKD treated with Klotho have shown improved phosphate handling and less vascular calcification (Hu et al., 2017; Hum et al., 2017). While not yet available clinically, Klotho-targeted therapies are being explored for CKD, which in theory would help “reset” the FGF23-Klotho axis toward normal.

## Clinical implications and therapeutic perspectives in ADPKD

FGF23 elevation in ADPKD has several important clinical implications, spanning its role as a biomarker of disease activity/progression and as a factor in systemic complications.

### Diagnostic marker of disease burden

Because FGF23 can rise early in ADPKD, it may serve as a marker of underlying disease burden that is not captured by GFR or serum creatinine (Pavik et al., 2012). An ADPKD patient might have normal kidney function yet significant cystic changes and hepatic involvement that lead to high FGF23. Bienaimé et al. demonstrated that FGF23 levels correlated with liver cyst severity (Bienaimé et al., 2018), suggesting FGF23 as a proxy for extrarenal (liver) cyst involvement. Thus, FGF23 could be explored as a non-invasive biomarker to identify patients with extensive cystic disease who might benefit from more aggressive therapy or monitoring, even before kidney function declines. It might also help distinguish ADPKD from other causes of CKD in cases where the diagnosis is uncertain.

### Prognostic indicator for progression

Growing evidence links higher FGF23 levels in ADPKD to more rapid disease progression. A notable study (HALT-PKD and others) found that ADPKD patients with higher baseline FGF23 had faster increase in total kidney volume (TKV) and more rapid decline in GFR over time (Chonchol et al., 2017). El Ters et al. reported that serum FGF23 was an independent prognostic biomarker for renal enlargement and function loss, as well as mortality, in early-stage ADPKD (El et al., 2021). This means FGF23 might help stratify patients by risk.

### Cardiovascular and systemic effects

ADPKD patients, like CKD patients, suffer cardiovascular issues even before ESRD. High FGF23 in ADPKD likely contributes similarly to cardiac remodeling and vascular dysfunction as it does in other CKD. Studies have observed that ADPKD patients with elevated FGF23 have greater left ventricular mass and arterial stiffness (Righini et al., 2024), paralleling CKD findings. Additionally, ADPKD has unique bone metabolism aspects: these patients often exhibit an adynamic bone disorder early in CKD (Righini et al., 2024; Gitomer et al., 2021). FGF23 excess, along with low 1,25D and perhaps elevated sclerostin, might contribute to this low bone turnover state. However, interestingly, ADPKD patients on dialysis do not have a higher fracture risk than other dialysis patients (Gitomer et al., 2021), suggesting some differences in bone quality. Nonetheless, systemically controlling FGF23 in ADPKD could be beneficial for reducing cardiovascular risk and improving overall mineral and bone health.

### Therapeutic implications

Ultimately, an ADPKD patient who develops advanced CKD will be subject to the same potential therapies for high FGF23 as any CKD patient. While there is no direct anti-FGF23 therapy in ADPKD, several interventions in ADPKD might indirectly influence FGF23 levels and effects: Tolvaptan, a vasopressin V2 receptor antagonist, is an approved disease-modifying treatment for ADPKD that slows cyst growth and preserves renal function (Gittus et al., 2025). By slowing the progression of CKD in ADPKD, tolvaptan may indirectly modulate FGF23 trajectorie. Additionally, by reducing cyst burden and possibly mitigating some aspects of cyst-induced injury, tolvaptan could reduce some stimuli for FGF23 production.

## Comparative insights across AKI, CKD, and ADPKD

Despite their differing etiologies, AKI, CKD, and ADPKD all exhibit elevated FGF23, yet the mechanisms, timing, and clinical implications vary markedly. Table 1 provides an overview of the key contrasts and commonalities in FGF23 biology among AKI, CKD, and ADPKD. In AKI, FGF23 rises abruptly in response to acute inflammation, ischemia, and metabolic stress, serving as a transient but sensitive biomarker of injury severity. CKD is characterized by a gradual, sustained FGF23 increase driven by phosphate retention, Klotho deficiency, and chronic inflammation, often correlating with cardiovascular risk and poor outcomes. In contrast, ADPKD features disproportionately high FGF23 levels even at preserved GFR, likely due to ectopic production (e.g., liver, cysts), early tubular dysfunction, and Klotho resistance. These differences highlight FGF23 as a shared indicator of renal stress, yet one whose regulation and clinical value are highly disease-specific.

**TABLE 1:** Summary of FGF23 regulation and implications in AKI vs. CKD vs. ADPKD. This table highlights the different triggers of FGF23 elevation, the timing and magnitude of changes, and the clinical significance in each condition.

Aspect	AKI	CKD	ADPKD
Expression kinetics	Rapid surge, within hours to days of injury; peaks early and may decline with recovery (if AKI reverses)	Gradual, exponential rise as kidney function declines; begins in early CKD (stage 2–3) and can reach 100–1,000× normal by ESRD. Persistent high plateau in advanced stages	Disproportionately elevated early, even with preserved GFR. Rises ahead of significant kidney failure; continues increasing as cystic disease progresses. Levels high relative to CKD stage (often 2–3× higher than non-ADPKD CKD at same GFR)
Primary sources of FGF23	Bone (osteocytes) is main source, stimulated by acute factors Possible extra-osseous production: Liver and injured kidney	Bone remains principal source (chronic osteocyte stimulation by phosphate, PTH, etc.). Little to no normal hepatic production. Some local kidney/tumor production in certain contexts (e.g., calcitriol-activated tubules) but systemic levels mainly from bone	Bone plus ectopic sources. Bone contributes, but cystic organs produce FGF23: Liver cysts and kidney cyst epithelium
Pathophysiological drivers	Inflammation (cytokines) – IL-6, IL-1β, TNF-α acutely upregulate FGF23 Ischemia/hypoxia – kidney injury releases G-3-P and induces HIF-1α, stimulating FGF23 Reduced clearance Klotho loss – acute shedding lowers Klotho, possibly increasing FGF23 (feedback due to resistance) Use as trigger: High FGF23 could prompt earlier RRT or more aggressive AKI support, hypothesized but not yet standard practice	Phosphate retention – declining GFR causes phosphate buildup, directly driving FGF23 secretion Klotho deficiency – chronic loss leads to FGF23 resistance and compensatory overshoot Secondary hyperparathyroidism – high PTH and low calcitriol stimulate FGF23 Chronic inflammation – persistent IL-6, TNF in CKD augment FGF23 production Iron deficiency/EPO – anemia of CKD and ESA use can increase FGF23	Cystic disease milieu Local hypoxia in large cysts (kidney & liver) triggers HIF-1α and FGF23 expression Cyst expansion & injury cause inflammation, cytokines Ectopic production – polycystic liver and renal cyst cells actively produce FGF23 Early Klotho downregulation – cystic kidneys have low Klotho, causing FGF23 resistance & excess output Tubular phosphate handling defects – “renal phosphate leak” in ADPKD leads to relative hypophosphatemia, which might paradoxically stimulate FGF23 (or reflect FGF23 action with resistance) Genetic factors – polycystin mutations may directly alter signaling pathways (Wnt/β-catenin, cilia) in osteocytes and tubules that modulate FGF23
Diagnostic value	Early AKI marker: FGF23 often rises before creatinine, indicating AKI onset or severity Prognosis: Extremely high FGF23 in AKI correlates with higher mortality and greater risk of transition to CKD	CKD progression and outcome predictor: FGF23 is a strong independent predictor of ESRD and mortality in CKD. Higher FGF23 in moderate CKD signals faster GFR decline and greater CV event risk. More sensitive than phosphate or PTH in early CKD. Rarely measured clinically yet, but a promising risk marker for CKD staging	Biomarker of cystic disease burden: Elevated FGF23 in early-stage ADPKD may reflect high total kidney volume or severe polycystic liver involvement Prognostic marker: High FGF23 is associated with faster kidney growth, quicker GFR decline, and greater risk of reaching kidney failure
Systemic effects	Primarily relevant in severe AKI cases: FGF23 surge can contribute to acute cardiac dysfunction. However, short duration limits chronic effects	Clear cardiovascular toxicity from chronically high FGF23: Promotes LVH, arterial stiffness, hypertension. Raises risk of heart failure and arrhythmias Anemia and immune dysregulation. Vascular calcification Secondary hyperparathyroidism (PTH resistance) and bone turnover alterations (renal osteodystrophy)	Similar systemic impacts to CKD once FGF23 is high: ADPKD patients with high FGF23 exhibit LVH and elevated hypertension Bone metabolism: ADPKD often shows adynamic bone disease in early CKD – high FGF23 with low 1,25D might be a factor Overall, systemic effects in ADPKD align with CKD effects (cardiac and bone), though onset may be earlier in the course
Therapeutic approaches	Largely experimental/supportive: No specific FGF23-targeted therapy yet. Focus is on treating the underlying AKI cause phosphate management Potential future strategies: IL-6 blockade, Klotho therapy, FGFR4 inhibitors	Well-established interventions (indirect): Dietary phosphate restriction & phosphate binders Vitamin D analogs and calcimimetics (cinacalcet/etelcalcetide) Iron supplementation HIF-stabilizers Dialysis	ADPKD-specific measures Tolvaptan: Slows cyst growth and CKD progression, thereby likely attenuating the steep rise of FGF23 that comes with later-stage CKD

AKI, acute kidney injury; CKD, chronic kidney disease; ADPKD, autosomal dominant polycystic kidney disease; FGF23 = fibroblast growth factor 23; GFR, glomerular filtration rate; PTH, parathyroid hormone; IL-6, interleukin-6; TNF-α = tumor necrosis factor α; G-3-P = glycerol-3-phosphate; HIF-1α = hypoxia-inducible factor 1α; FGFR4 = FGF, receptor 4; EPO = erythropoietin; ESA, erythropoiesis; CV, cardiovascular; RAAS, renin-angiotensin-aldosterone system; LVH, left ventricular hypertrophy; RRT, renal replacement therapy.

## Controversies and future directions

While FGF23 is increasingly recognized as a biomarker and potential mediator of kidney disease progression, several controversies remain unresolved. One major debate is whether elevated FGF23 is simply a compensatory response to phosphate imbalance or a direct contributor to disease pathology, especially in cardiovascular and fibrotic complications. For instance, some experimental studies suggest that FGF23 promotes left ventricular hypertrophy and renal fibrosis via FGFR4 signaling, but it remains unclear whether targeting FGF23 itself is beneficial or harmful, given its physiological role in mineral homeostasis. Another point of contention lies in ADPKD, where the source of FGF23 elevation—bone vs. liver vs. cysts—varies across studies, and the clinical utility of FGF23 as a disease activity marker is still being validated. Additionally, the use of FGF23 as a therapeutic target raises concerns: neutralizing FGF23 may correct its excess but precipitate hyperphosphatemia or vascular calcification, particularly in advanced CKD. These uncertainties underscore the need for further mechanistic and interventional studies to clarify when FGF23 is a marker, a mediator, or a modifiable risk factor in different renal pathologies.

## Conclusion

FGF23 acts as a shared yet heterogeneously regulated biomarker across AKI, CKD, and ADPKD. In AKI, its rapid surge reflects acute stress and may aid early diagnosis; in CKD, a sustained rise contributes to long-term complications; in ADPKD, early and disproportionate elevations point to disease-specific drivers. These patterns underscore the dual role of FGF23—as both a marker and a potential contributor to pathology. A better understanding of its context-specific regulation could support more targeted diagnostics and therapies across the spectrum of kidney diseases.
